# Geochemical and remote sensing integrated with satellite gravity data of Darhib and Atshan talc deposits, South Eastern Desert, Egypt

**DOI:** 10.1038/s41598-023-31398-x

**Published:** 2023-06-05

**Authors:** El Saeed R. Lasheen, Waheed H. Mohamed, Mahmoud H. Elyaseer, Mohamed A. Rashwan, Mokhles K. Azer

**Affiliations:** 1 Geology Department, Faculty of Science, Al-Azhar University, P.O. Box 11884, Cairo, Egypt; 2 National Research Centre, Cairo, Egypt

**Keywords:** Environmental sciences, Solid Earth sciences

## Abstract

The current contribution conducted new geochemical, remote sensing integrated with gravity detailed studies of talc deposits to identify the talc protolith as well as its extension, depth, and structures. There are two examined areas, distributed from north to south, Atshan and Darhib and both belong to the southern sector of the Egyptian Eastern Desert. They occur as individual lenses or pocket bodies in ultramafic-metavolcanics following NNW-SSE and E-W shear zones. Geochemically, among the investigated talc, Atshan samples have high contents of SiO_2_ (av. 60.73 wt.%), and higher concentrations of transition elements such as Co (av. 53.92 ppm), Cr (781 ppm), Ni (av. 1303.6 ppm), V (av. 16.67 ppm), and Zn (av. 55.7 ppm). Notably, the examined talc deposits contain low contents of CaO (av. 0.32 wt.%), TiO_2_ (av. 0.04 wt.%), SiO_2_/MgO (av. 2.15), and Al_2_O_3_ (av. 0.72 wt.%), which is comparable with ophiolitic peridotite and forearc setting. False color composite (FCC), principal component analysis (PCA), minimum noise fraction (MNF), and band ratio (BR) have been used to distinguish talc deposits in the investigated areas. Two new proposed band ratios were created to separate talc deposits. FCC band ratios (2/4, 4/7, 6/5) and (4 + 3/5, 5/7, 2 + 1/3) have been derived to focus on talc deposits in two case studies, Atshan and Darhib areas. The application of regional, residual, horizontal gradient (HG), and analytical signal (AS) techniques to gravity data are used in interpreting the structural directions of the study area. The analysis of this technique displays several notable faults trending in NW–SE, NE–SW, NNW–SSE, and E–W directions. Two techniques of gravity depth calculation were applied in the study areas, namely source parameter image (SPI), and Euler deconvolution (EU). The analysis of these techniques reflects that the depth of subsurface sources ranges between 383 and 3560 m. Talc deposits may be attributed to greenschist facies metamorphism or to a magmatic solution that is (associated with granitic intrusions) interacted with the surrounding volcanic rocks forming metasomatic minerals.

## Introduction

The economic significance of talc is ascribed to it is variable industrial applications due to it has distinctive characteristics. Therefore, recent studies have been focused on talc deposits^1–4^. It is widely used in paints, ceramics, foods, rubber, electrical cable, cosmetics, and prescription drugs depending on its purity^1,2^. It’s widely used for the hexavalent chromium adsorption process, which acts as a clarifier for wastewater^5^.

Talc deposits are widely, distributed in the central and southern sectors of the Egyptian Eastern Desert, associated with ophiolitic and metavolcanic rocks^6–9^. Ophiolites are slices of the oceanic lithosphere, that are thrusted onto the continental plates, helping to recognize the tectonic processes in the mantle section^7,8,10,11^. Serpentinites, carbonatized serpentinites, talc-carbonates, and listwaenite (carbonate-rich, silica-carbonate, and birbirites) rocks are the main alteration products of the ophiolitic ultramafic (peridotites/dunite) fragments due to interaction with CO_2_- and SiO_2_-rich fluids^7,8,10,11^. Pure talc mineralization or contamination with carbonate minerals that are widely occurring along fault planes and/or shear zones^6,7,10^. Egyptian talc mineralization is massive, moderately to strongly schistose (smooth surface), and fine-grained, reflecting a low to medium grade of metamorphism (greenschist-lower amphibolite facies)^7,8,10,11^. Ultramafic rocks are enriched with magnesium and iron silicate minerals. Carbonation processes are taken place by the hydrolysis of these minerals through the eviction of Si and combination of these cations with carbonates^7,9,12^.

Economic talc deposits that are mined such as Darhib, Atshan, and Wadi Allaqi regions from thirty five recorded talc deposit occurrences in Sinai and the Eastern Desert^13^. They produced approximately 12,924 and 172,181 tonnes of talc in 2011 and 2015, respectively^3,14^.

The gravity technique is one of the geophysical tools applicable to a lot of exploration. The gravity technique includes determining the location of subsurface density changes by measuring the earth's gravitational field at certain points on the earth's surface^15^.

The current work aims to investigate the geological and geochemical detailed studies of the Atshan and Darhib areas to deduce the origin of talc deposits. Remote sensing integrated with gravity data was conducted to reveal the talc extension and its depth, as well as to determine the dominant surface and subsurface structure controlling the distribution of talc deposits.

## Geological background

During the collision of the East and West supercontinents (Gondwana), the Neoproterozoic northern extension (Arabian Nubian Shield, ANS) of the Mozambique belt was formed by the accretion of arcs and other continents^16–19^. The ANS represents one of the best-preserved juvenile crusts that provides information about the nature and sources of the widespread rocks during the East African Orogeny^20–23^. The ANS crustal growths comprise remnants of ophiolitic rocks and arc assemblages (820–720 Ma), variable collisional rocks (630–620 Ma), and post-collisional granitic rocks (620–580 Ma), forming three tectonic events^14,24^. The Egyptian Eastern Desert forms the northern part of the ANS, which can be subdivided into northern, central, and southern sectors^25^. Talc and magnesite represent the dominant mineralization associated with ultramafic (ophiolitic) rocks^8,18,26^.

**Figure 1:**
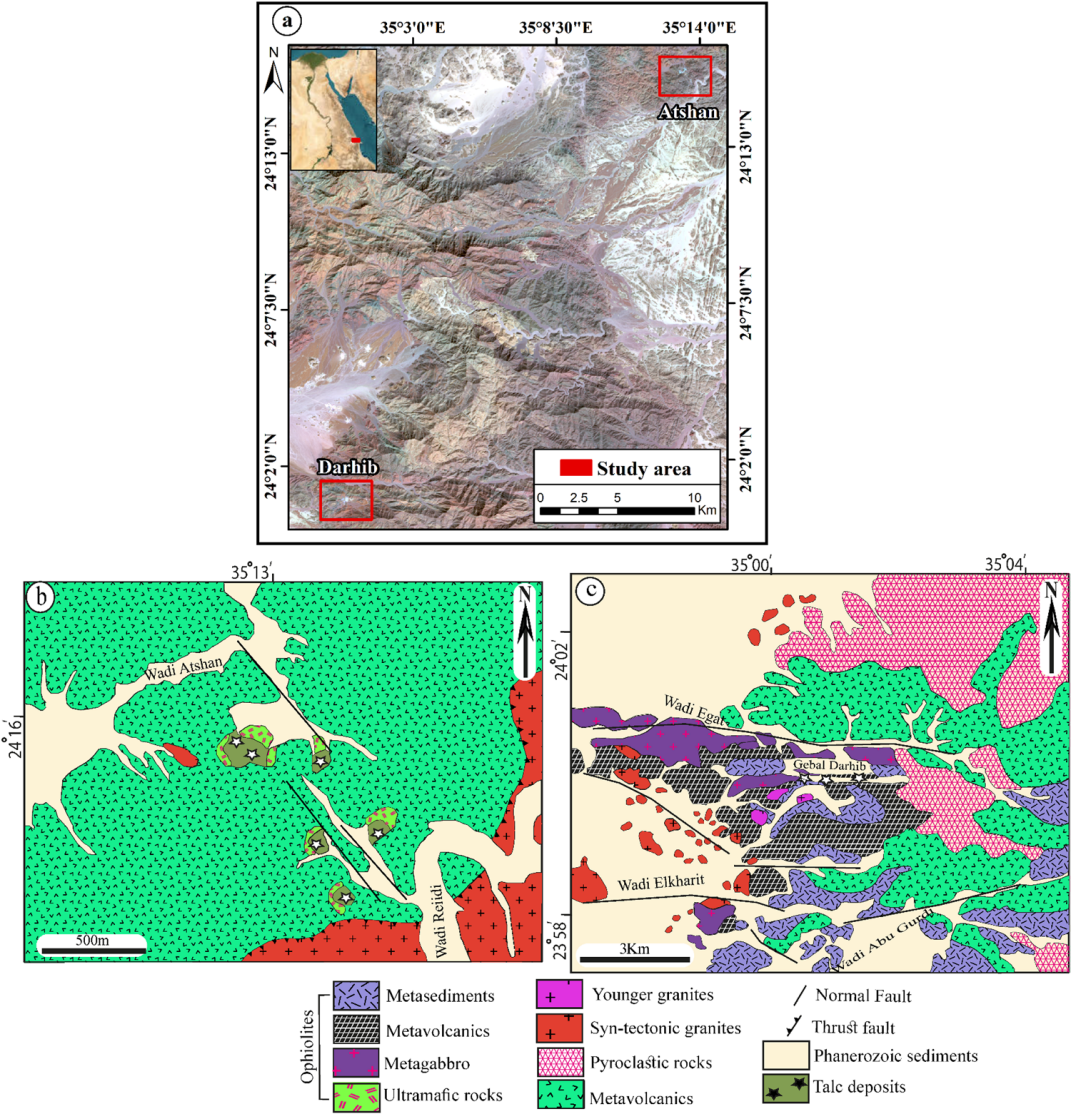
(**a**) Location map of Atshan and Darhib areas, South Eastern Desert, Egypt (using Arc GIS 10.4 and ENVI 5.3. The acquisition date of Landsat-8 image: September 8, 2021, with path 173 and row 43. Source Landsat-8: http://earthexplorer.usgs.gov. (**b**) Detailed geologic map of Atshan area^13^ (using Adobe Illustrator program CS5); and (**c**) Detailed geologic map of Darhib area^4^ (using Adobe Illustrator program CS5).

**Figure 2:**
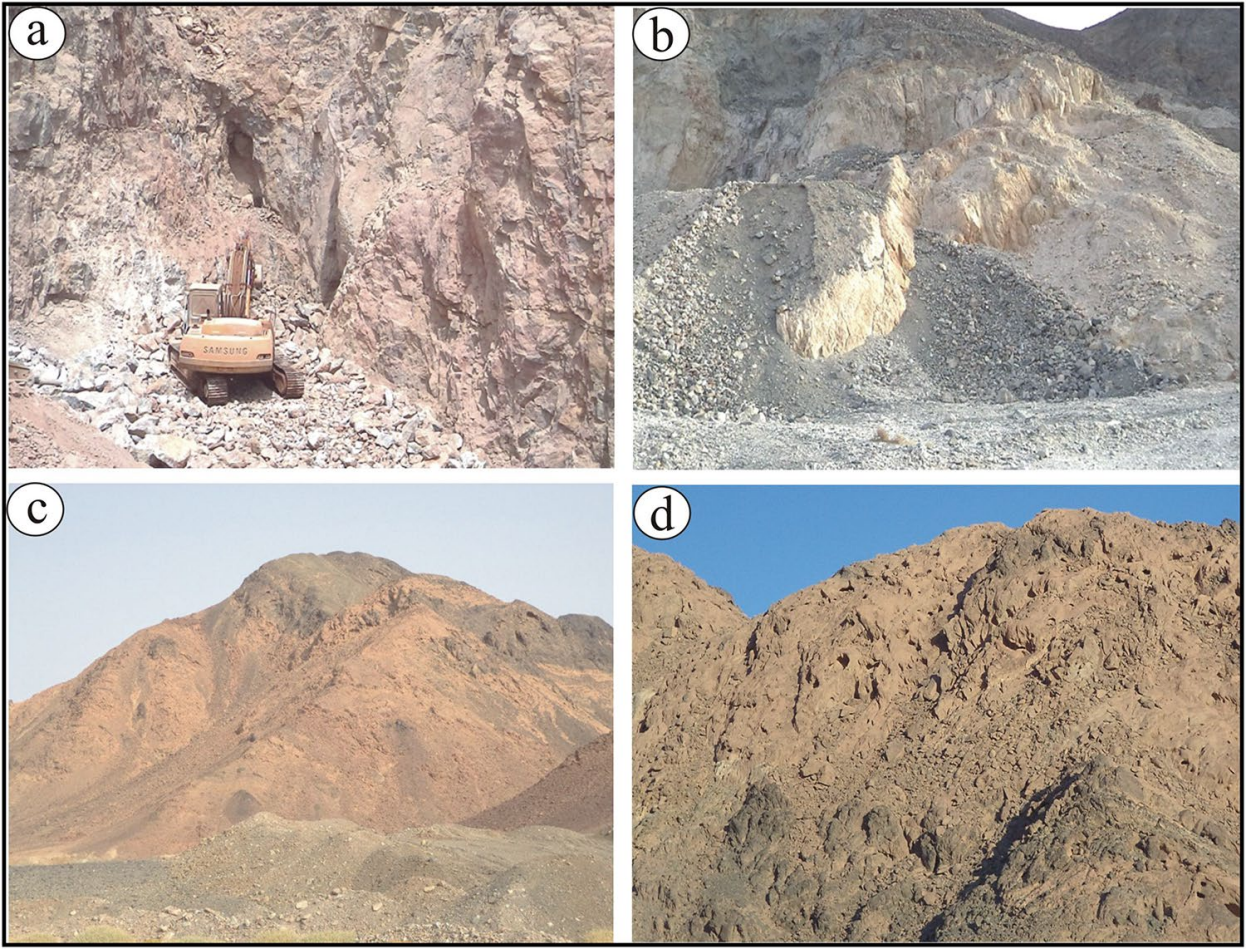
Field photographs of Atshan and Darhib areas, South Eastern Desert, Egypt: (**a**,**b**) Pockets of talc deposits enclosed in ultramafic rocks; and in (**c**,**d**) metavolcanics.

The two examined areas are distributed from north to south; Atshan and Darhib and both belong to the southern sector of the Egyptian Eastern Desert (Fig. 1a). Atshan area lies in the Hamata district, about 18 km from the Red Sea. The dominant lithological units are ultramafics (serpentinites), metavolcanic, and Reiidi syn-tectonic granites (Fig. 1b). These ultramafic rocks are extensively transformed to talc, tremolite, and carbonates, particularly along the longest NNW-SSE (> 1000 m long) and E–W (700 m long) shear zones or fault planes. Metavolcanics, include both mafic and felsic types, which represent the dominant rock units in the Atshan area, and are intruded by syn-tectonic granites (Wadi Reiidi). Talc-rich rocks are five individual lenses or pocket bodies in ultramafics and metavolcanics along the mineralized shear zones in the Atshan area (Fig. 2a,b). Some of these pockets sporadically belong to NNW-SSE shear zone along the contact between metavolcanics and serpentinites. Small sulfide bodies are recorded within the talc + tremolite bodies that are ascribed to metamorphism^4,13^. Atshan mine represent the largest talc producer in the period from 1962 to 1992 with about 60,000 tonnes from the estimated talc reserve^13^.

On the other hand, the exposed lithological rock units in the examined Darhib area are; ophiolitic, metavolcanics, syn-tectonic granites, and younger gabbro rocks (Fig. 1c). The ophiolitic rocks, cover small areas, which are represented by metagabbros and metavolcanics that are enclosed in metasediments. Talc deposits as well as carbonate- and tremolite-bearing rocks are enclosed by metavolcanics (mafic to felsic) certainly along fault planes and shear zones (Fig. 2c,d). The main shear zone extends in the E-W direction. This talc mine represents one of the largest source of high-grade in Egypt that is enriched with the disseminated sulfide minerals^13^.

From the previous, it is noticeable that the talc deposits are restricted along the predominant shear zones and fault planes, reflecting the role of metamorphism.

## Material and methods

### Field and petrography

**Figure 3:**
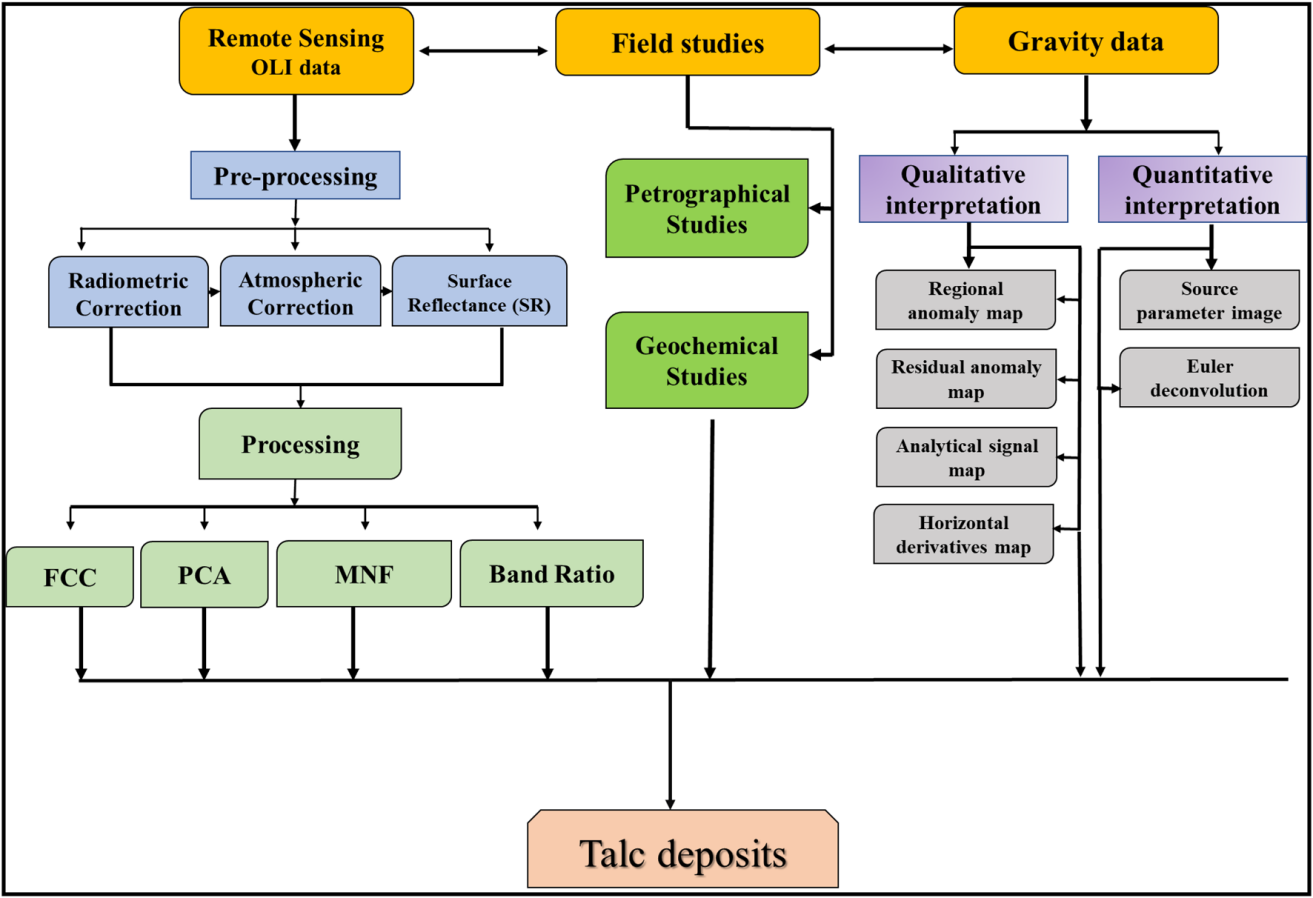
Flowchart summarized the methodology of the current work.

More than twenty samples have been collected from both examined areas (Fig. 3). Twelve samples of talc deposits from the two studied areas are prepared as thin sections, and their preliminary mineralogical compositions were detected by using a polarizing microscope (Fig. 3) at the Rocks Lab, Faculty of Science, Al-Azhar University.

### Geochemistry

Bulk rock geochemistry (major and trace) of twelve representative talc samples from the two studied were analyzed at the National Research Centre (Fig. 3). All analyzed samples were prepared as a bead with a 1 gm sample/10 gm flux ratio (66% of lithium tetraborate: 34% of Lithium metaborate) at 1150 °C in an electroconductive furnace. ASTM E-1621 and ASTM D-7348 are the main standard guides used in the analysis. PANalytical 2005 and Axios Advanced are used to detect the concentration of elements. The measurement precisions of the analyses were ± 5% and ± 10% for major and trace elements, respectively.

### Remote sensing data

Landsat-8 images of the study area have been used in this study (Fig. 3). The utilization of Landsat-8 data rather than Aster data in the identification of talc deposits in this study was primarily due to the limited extent of the study area. Landsat-8 is equipped with two sensors, the Operational Land Imager (OLI) and the Thermal Infrared Sensor (TIRS). OLI is represented by nine bands, but only two bands are provided by TIRS data. The scene that covers the investigated area was acquired on September 8, 2021, with path 173 and row 43. The data used are georeferenced to WGS 84 zone 36 N, UTM.

Then, we carried out the atmospheric correction using the fast line of sight atmospheric analysis of spectral hypercubes (FLAASH) technique^27^, then resized data to the extent of the study area. These procedures were performed utilizing ENVI 5.3 software. Image processing methods i.e., band combination (FCC), band ratio (BR), principal component analysis (PCA), and minimum noise fraction (MNF) were used to discriminate between different lithological rock units with emphasis on talc deposits.

### Gravity data

Gravity anomalies are generated from the available Earth global gravity models (EGM 2008) and DTU10 and contain 1 × 1 resolution terrain correction produced from the ETOPO1 model that evaluates the contribution of most surface masses (atmosphere, land, oceans, inland seas, lakes, ice caps, and ice shelves). These products were computed utilizing a spherical harmonic technique and theoretical improvements for accurate computations on a global scale. (http://bgi.obs-mip.fr/data-products/outils/wgm2012-maps-visualizationextraction/). Using Oasis Montag software version 8.3, various techniques such as regional, residual, analytical signal, horizontal derivatives, source parameter images, and Euler deconvolution were applied to gravity data to determine the subsurface structure and calculate the depth of sources (Fig. 3).

## Ethical approval and consent to participate

This article does not contain any studies with human participants or animals performed by any of the authors.All authors are agreed to be as authors in the current order in this manuscript version.

## Results

### Petrographical investigations

The preliminary minerals of two talc deposits have been detected using a polarizing microscope.

**Figure 4:**
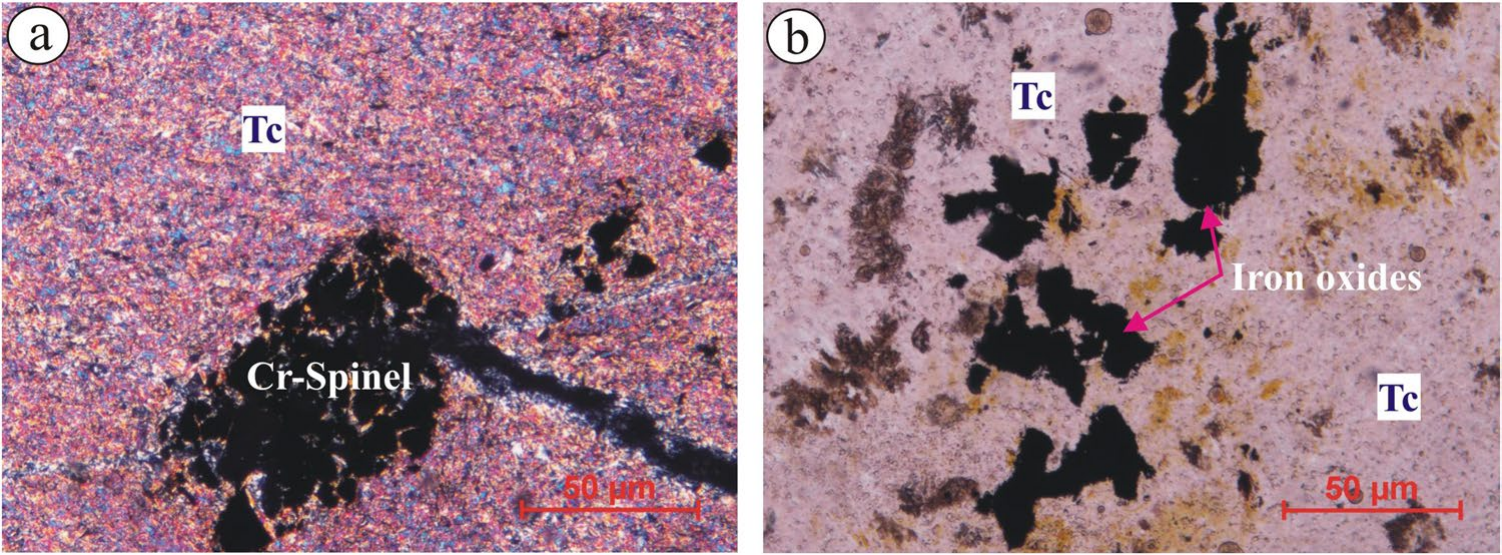
Photomicrographs (by using polarizing microscope (Olympus X53)) of Atshan and Darhib areas, South Eastern Desert, Egypt: (**a**) Fine-grained talc (Tc) minerals occur as shreds in Atshan area, and (**b**) abundance of opaque minerals (iron oxides) in Darhib area.

Atshan talc deposits are fine-grained than talc of Darhib deposits. It’s composed dominantly of talc mineral (> 95 vol. %), with subordinate (minor) minerals such as tremolite, serpentine carbonates, and opaques. It ranges in colour from pale green to greenish grey. Talc deposits exhibit lepidoblastic texture. It’s mostly distributed as shreds and dense microcrystalline fibrous grains (Fig. 4a). It’s noticed that serpentine minerals are abundant in association with talc minerals, reflecting ultramafic protolith. Spare opaque minerals are observed with anhedral to euhedral crystals of Cr-spinel, Fe-Ti oxides, and sulfide (pyrite).

Likewise, fine-grained Darhib talc deposits have the same mineralogical constituents as Atshan. Talc deposits exhibit lepidoblastic texture with a parallel arrangement of sheared and foliated talc and tremolite minerals. Acicular tremolite and carbonate patches occur as disseminated crystals embedded in a very fine matrix of talc. Opaque minerals are commonly medium-grained and euhedral fractured pyrite and/or Fe-Ti oxides (Fig. 4b).

### Geochemistry

**Figure 5:**
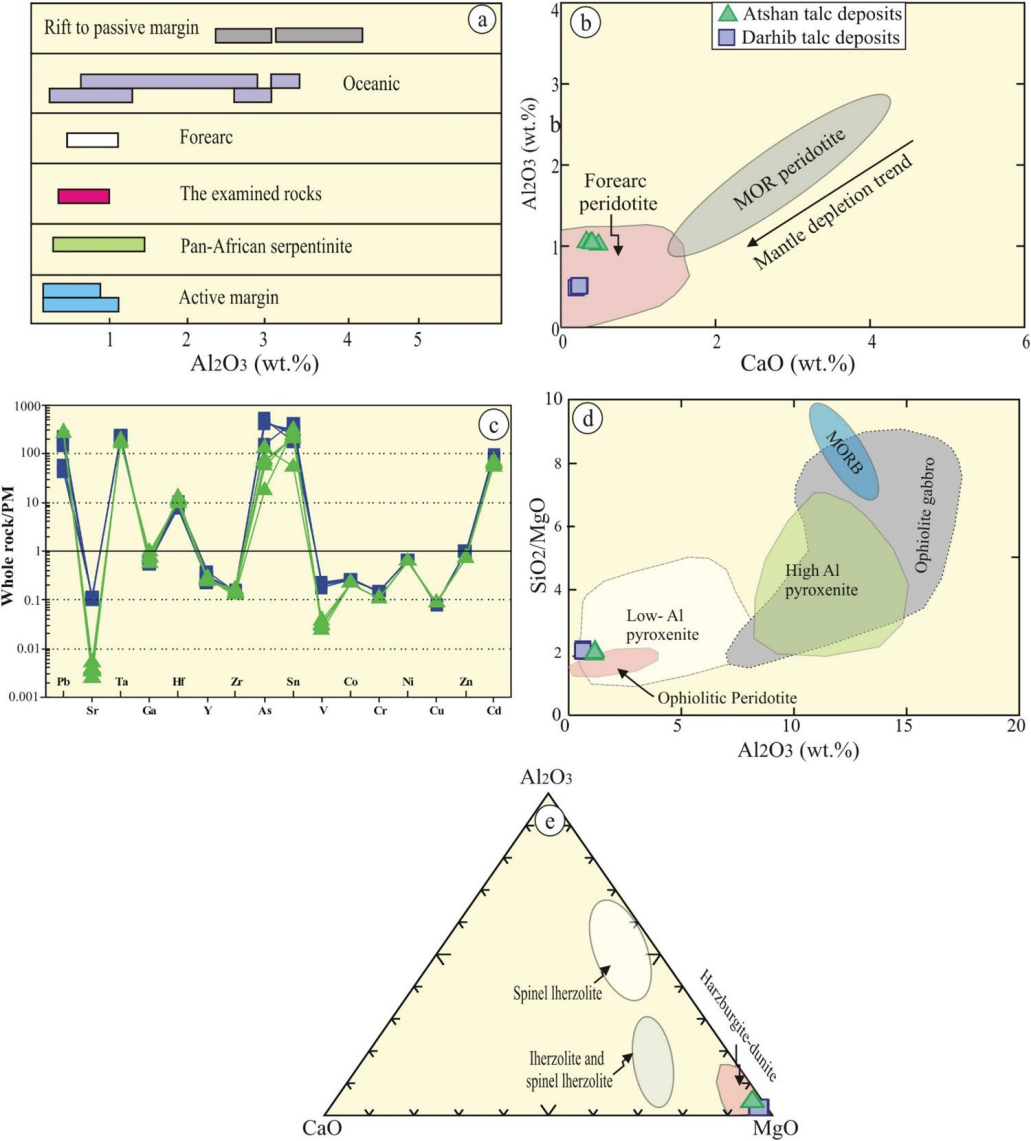
Whole rock diagrams (by using Coreldrow program version 2012): (**a**) Al_2_O_3_ of the examined samples are compared to forearc, Pan-African serpentinites^45^ and others^57^; (**b**) Al_2_O_3_ vs. CaO^58^; (**c**) Trace elements normalized to primitive mantle^29^; (**d**) SiO_2_/MgO vs. Al_2_O_3_ binary diagram^28^; and (**e**) CaO–Al_2_O_3_–MgO diagram^59^.

The bulk rock (major (wt.%) and trace (ppm) elements) of the examined talc deposits are given in Table 1. Among the investigated talc, Atshan samples have high contents of SiO_2_ (av. 60.73 wt.%), and higher concentrations of transition elements such as Co (av. 53.92 ppm), Cr (781 ppm), Ni (av. 1303.6 ppm), V (av. 16.67 ppm), and Zn (av. 55.7 ppm). In addition, Atshan samples possess high concentrations of chalcophile elements such as As (av. 10.86 ppm). On the contrary, Darhib samples have high contents of MgO (av. 27.95 wt.%) and semi-volatile elements such as Pb (av. 20.78 ppm) relative to Atshan samples. Both of the examined samples have low contents (less than unity) of TiO_2_, Al_2_O_3_, Na_2_O, Cr_2_O_3_, CaO, and MnO, reflecting the residual nature of their protolith, which are similar to forearc peridotite and Pan-African serpentinites^8,11,26,28^ (Fig. 5a,b).

**Table 1:** Major (%) and trace elements (ppm) of Atshan and Darhib talc deposits.

	Atshan						Av	Darhib						Av
SiO_2_	60.49	61.20	60.47	60.72	60.84	60.68	60.73	59.42	58.55	59.58	59.18	58.98	59.07	59.13
TiO2	0.01	0.01	0.01	0.01	0.01	0.01	0.01	0.06	0.09	0.06	0.07	0.07	0.07	0.07
Al2O3	0.43	0.46	0.46	0.45	0.46	0.46	0.45	1.00	0.97	1.00	0.99	0.98	0.98	0.98
Fe2O3	5.47	5.28	5.20	5.32	5.24	5.25	5.29	5.49	5.22	5.41	5.37	5.36	5.34	5.36
Cr2O3	0.22	0.21	0.20	0.21	0.21	0.20	0.21	0.32	0.26	0.32	0.30	0.29	0.29	0.29
Na2O	0.15	0.17	0.17	0.16	0.17	0.17	0.16	0.09	0.09	0.09	0.09	0.09	0.09	0.09
MgO	27.96	27.36	28.16	27.83	27.76	27.92	27.83	27.55	28.65	27.46	27.89	28.10	28.02	27.95
CaO	0.21	0.26	0.25	0.24	0.26	0.25	0.24	0.34	0.50	0.34	0.40	0.42	0.42	0.40
MnO	0.07	0.07	0.07	0.07	0.07	0.07	0.07	0.06	0.05	0.06	0.06	0.06	0.06	0.06
LOI	4.52	4.52	4.52	4.52	4.52	4.52	4.52	5.28	5.30	5.30	5.29	5.29	5.30	5.29
Total	99.53	99.54	99.50	99.52	99.52	99.51	99.52	99.62	99.67	99.62	99.63	99.64	99.64	99.64
Ag	1.00	0.90	0.95	0.95	0.93	0.94	0.94	0.90	3.20	2.05	2.05	2.43	2.13	2.13
As	7.80	13.30	10.55	10.55	11.93	11.01	10.86	6.70	0.90	3.80	3.80	2.83	3.61	3.61
Ba	–	–	–	–	–	–	–	–	–	–	–	–	–	–
Br	3.00	5.10	4.05	4.05	4.58	4.23	4.17	2.80	4.10	3.45	3.45	3.67	3.49	3.49
Cd	3.80	2.80	3.30	3.30	3.05	3.22	3.24	2.20	2.90	2.55	2.55	2.67	2.57	2.57
Co	52.70	54.90	53.80	53.80	54.35	53.98	53.92	46.10	46.00	46.05	46.05	46.03	46.05	46.05
Cr	779.70	782.20	780.95	780.95	781.58	781.16	781.09	564.60	573.00	568.80	568.80	570.20	569.08	569.08
Cu	4.90	4.60	4.75	4.75	4.68	4.73	4.73	5.10	5.20	5.15	5.15	5.17	5.15	5.15
Ga	2.30	2.20	2.25	2.25	2.23	2.24	2.24	3.90	2.20	3.05	3.05	2.77	2.99	2.99
Hf	2.90	2.10	2.50	2.50	2.30	2.43	2.46	2.80	3.60	3.20	3.20	3.33	3.23	3.23
Ni	1302.00	1304.90	1303.45	1303.45	1304.18	1303.69	1303.61	1201.10	1201.30	1201.20	1201.20	1201.23	1201.21	1201.21
Pb	17.00	6.50	11.75	11.75	9.13	10.88	11.17	21.10	20.50	20.80	20.80	20.70	20.78	20.78
Sn	28.40	11.40	19.90	19.90	15.65	18.48	18.96	7.00	20.20	13.60	13.60	15.80	14.04	14.04
V	14.50	18.40	16.45	16.45	17.43	16.78	16.67	6.20	3.90	5.05	5.05	4.67	4.97	4.97
W	32.90	33.40	33.15	33.15	33.28	33.19	33.18	23.90	27.50	25.70	25.70	26.30	25.82	25.82
Y	1.60	1.00	1.30	1.30	1.15	1.25	1.27	1.00	1.20	1.10	1.10	1.13	1.11	1.11
Zn	55.50	55.90	55.70	55.70	55.80	55.73	55.72	40.30	39.40	39.85	39.85	39.70	39.82	39.82
Zr	3.20	3.20	3.20	3.20	3.20	3.20	3.20	3.40	2.60	3.00	3.00	2.87	2.97	2.97
Rb	–	–	–	–	–	–	–	–	–	–	–	–	–	–
Sr	4.00	4.60	4.30	4.30	4.45	4.35	4.33	0.20	0.10	0.15	0.15	0.13	0.15	0.15
Ta	9.00	8.00	8.50	8.50	8.25	8.42	8.44	6.20	7.10	6.65	6.65	6.80	6.68	6.68
Nb	–	–	–	–	–	–	–	–	–	–	–	–	–	–

-, Not detected.

It’s noticeable from the primitive mantle normalized trace elements^29^ diagrams that the examined samples reveal clear depletion of incompatible elements such as Co, Cu, and Cr with Ni positive anomalies (Fig. 5c). Furthermore, the positive anomaly of semi-volatile elements such as Pb and strong the negative anomaly of LFSEs such as Sr are observed. In addition, clearly pronounced positive As, Sn, and Cd anomalies are attributed to the abundance of the sulfide minerals^4^.

### Lithological mapping

#### False color composite (FCC)

**Figure 6:**
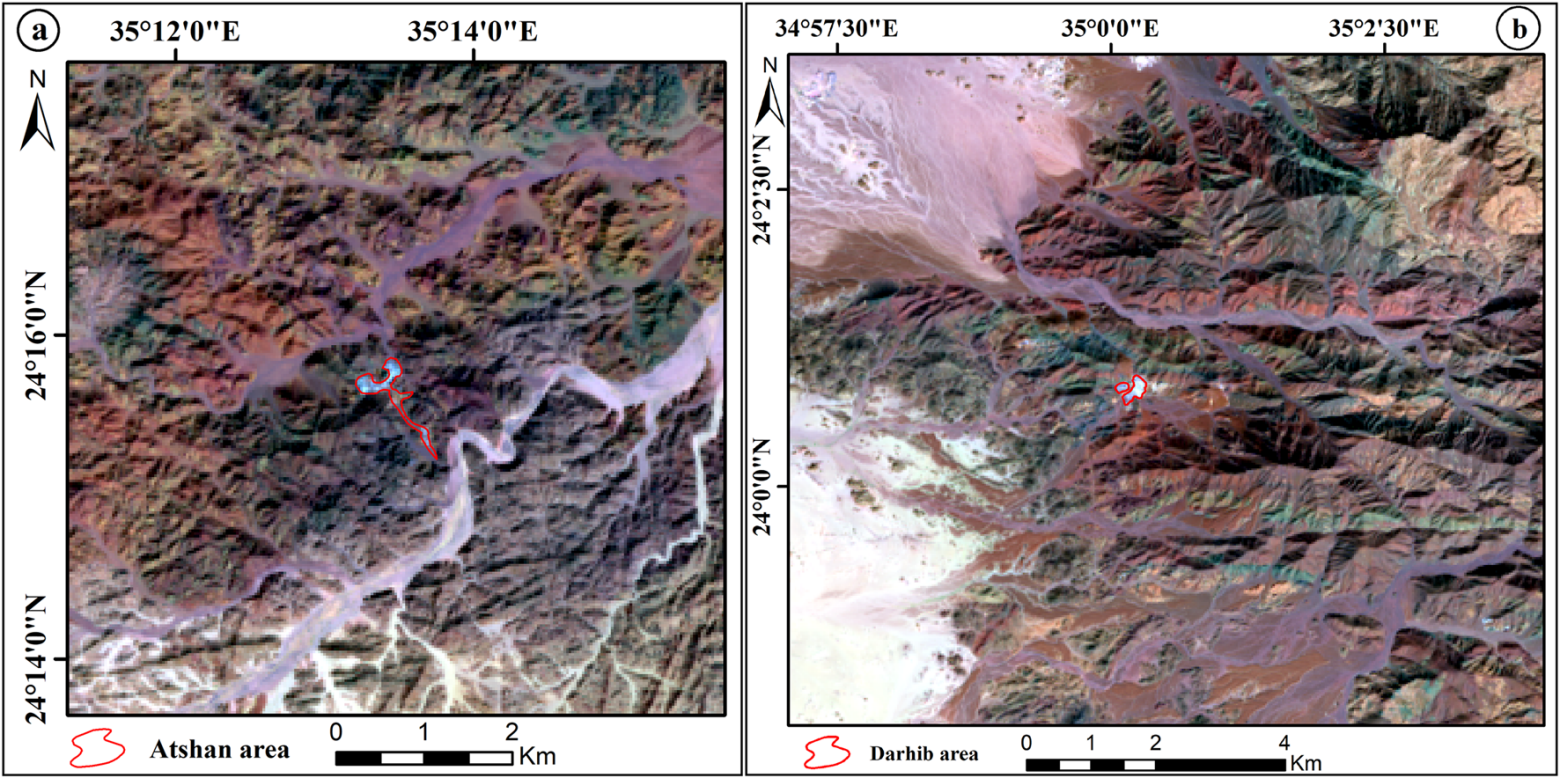
(**a**) Landsat-8 7, 5, 3 in RGB false color composite of Atshan area, and (**b**) Landsat-8 7, 5, 3 in RGB false color composite of Darhib area by using Arc GIS 10.4 and ENVI 5.3.

The Landsat-8 (OLI) data includes seven VNIR and SWIR spectral bands. To construct a color image using the data from these bands, just three bands are required in a band combination. The most effective band combinations are those that increase the intended target and include the most informative bands with the least amount of information redundancy and the fewest number of intercorrelated bands^30^. Several spectral bands of the OLI data were evaluated to get the best FCC images in the research region that exhibit the best lithological discrimination. The Optimum Index Factor (OIF) method has been used combined with RGB color composite using the ILWIS software. The OIF result analysis showed a different composite of OLI bands for enhancing different rock units of the research area (Table 2). These FCC are a result of the relationship between standard deviations and correlation coefficients of the data used^31^. The Landsat-8 (753) RGB distinguishes well between several lithological units of the Atshan and Darhib areas (Fig. 6a,b).

**Table 2:** The highest ranking of OIF results.

Rank	Band triplet	%
1	7,5,3	72.17
2	7,5,1	72.06
3	7,6,2	71.63
4	7,2,1	71.35
5	7,3,1	70.81
6	7,5,2	70.62

#### Principal component analysis (PCA)

The multivariate statistical methodology and dimensionality reduction technique used frequently with remote sensing data is the principal component analysis (PCA)^30^. PCA is used to produce bands that are uncorrelated to separate noise components and minimize the data's spectral dimensionality. The PCA band data are non-correlated and independent and are often more interpretable than the source data. Principal component analysis (PCA) is one of the most important techniques for lithological discrimination^32^. The principal component analysis transformation was done for the Landsat-8 VNIR and SWIR bands to obtain lithological data.

**Figure 7:**
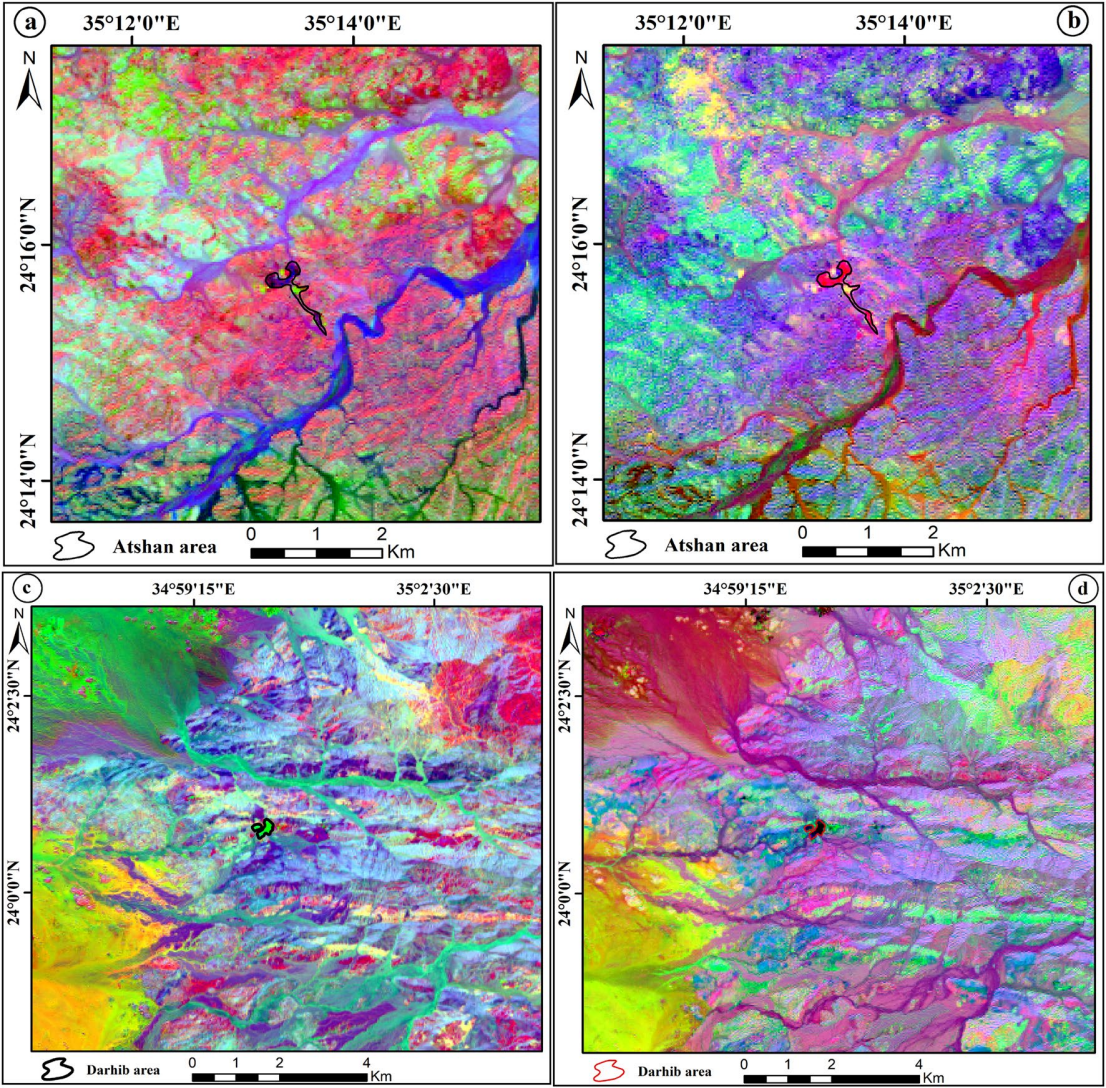
The FCC of principal component analysis: (**a**) Landsat-8 (RGB-PC1, PC2, PC3), (**b**) Landsat-8 (RGB-PC4, PC2, PC1) of Atshan area. (**c**) Landsat-8 (RGB-PC3, PC2, PC1), and (**d**) Landsat-8 (RGB-PC4, PC3, PC1) of Darhib area by using Arc GIS 10.4 and ENVI 5.3.

The eigenvalues of OLI data created using the PCA revealed that the first PCA contains the highest variance at 95.83%, and the second PCA band contains a second high variance at 2.95% (Table 3). Based on the eigenvector analysis, the better PCA bands are PC1, PC2, PC3, and PC4. The false color composite of PCA (PC1, PC2, PC3) in RGB discriminates talc deposits of the Atshan area with a dark blue color (Fig. 7a) while the FCC (RGB- PC4, PC2, PC1) differential the same deposits of this area by a deep pink (Fig. 7b). For talc deposits in the Darhib area, authors produced the color composite images (RGB- PC3, PC2, PC1, and RGB- PC4, PC3, PC1) to separate this deposit with black and lime color (Fig. 7c,d) respectively.

**Table 3:** The eigenvector values of PCA for Landsat-8 bands.

Eigenvector	Band 1	Band 2	Band 3	Band 4	Band 5	Band 6	Band 7	Eigenvalues %
PC 1	0.9318	− 0.3512	0.08086	− 0.0438	0.0002	− 0.0004	0.00142	95.83
PC 2	− 0.29157	0.8543	− 0.4064	− 0.1015	0.09809	0.00198	− 0.0049	2.95
PC 3	0.19939	− 0.2991	0.87965	− 0.2182	0.22019	0.02908	− 0.0056	0.66
PC 4	− 0.025	0.15422	− 0.1403	− 0.9642	− 0.1612	− 0.0116	0.01049	0.32
PC 5	− 0.0712	− 0.16038	− 0.1694	− 0.0889	− 0.75793	0.53086	− 0.1187	0.12
PC 6	0.03617	− 0.0867	0.07753	− 0.04854	− 0.5231	0.77822	− 0.3218	0.11
PC 7	0.00777	− 0.0169	0.0097	0.01441	− 0.0748	0.33403	− 0.93924	0.04

#### Minimum noise fraction transform (MNF)

**Figure 8:**
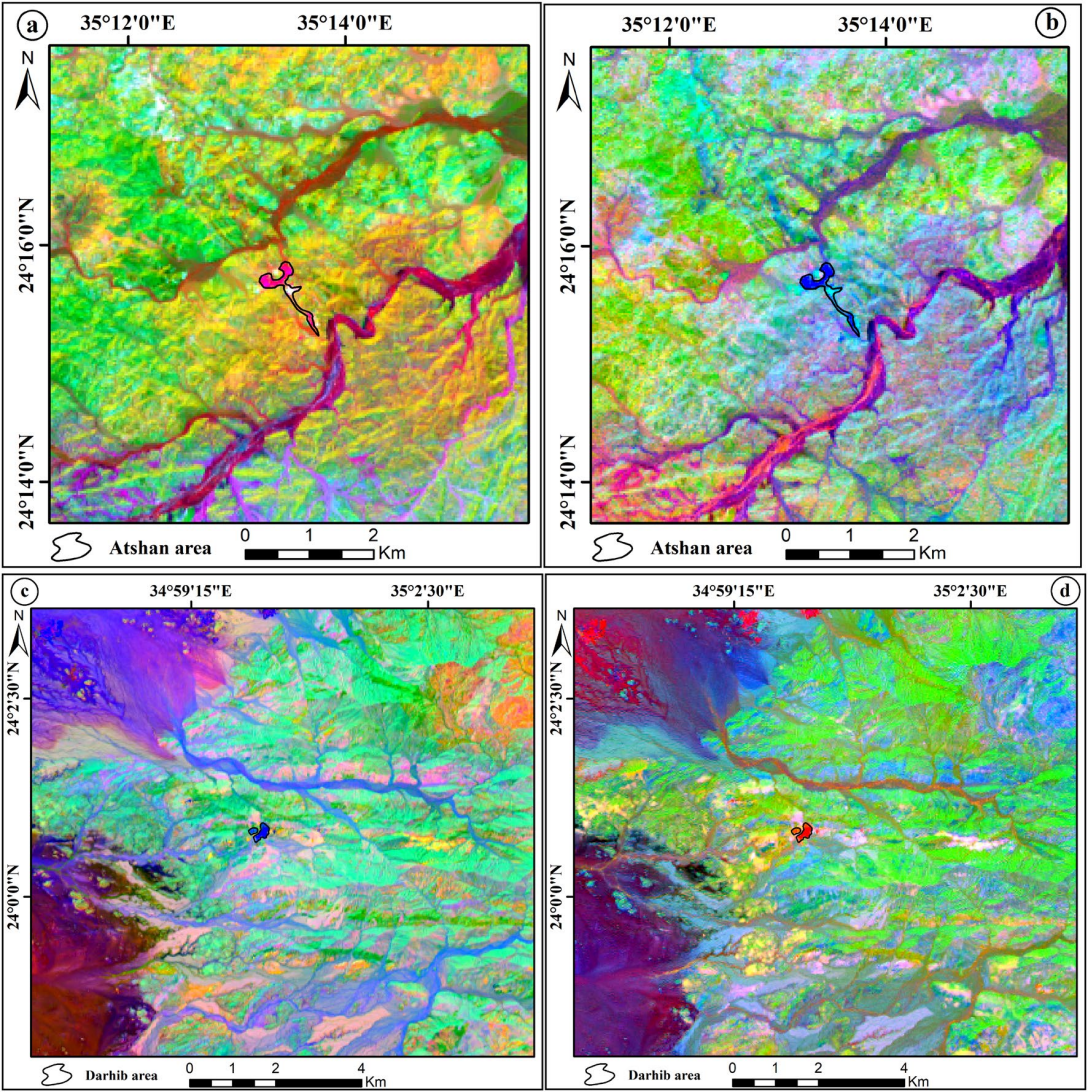
The FCC of Minimum noise fraction transform: (**a**) Landsat-8 (RGB-MNF3, MNF1, MNF2), (**b**) Landsat-8 (RGB-MNF4, MNF1, MNF3) of Atshan area. (**c**) Landsat-8 (RGB-MNF3, MNF1, MNF2), and (**d**) Landsat-8 (RGB-MNF4, MNF1, MNF3) of Darhib area by using Arc GIS 10.4 and ENVI 5.3.

The MNF transform technique is an algorithm consisting of successive data reduction operations, the first operation being based on an estimation of noise in the data as represented by a correlation matrix. This transformation decorrelates and rescales the noise in the data by making distinctions. The second operation takes the original correlation into account and creates a set of components containing weighted information about the variance in all bands of dataset^33^. MNF similar to principal component analyses, in that it also requires reducing residual noise from the spectral data as a first step, which makes it easier to choose prototype spectra. MNF transform separates the spectral bands with significant information contributing to the overall variance in the dataset from the bands that are dominated by noise^34^. The MNF results of this study show that the color composite of MNF bands MNF3, MNF1, MNF2; MNF4, MNF1, and MNF3 in RGB represents talc deposits of Atshan area by pink and blue color, respectively (Fig. 8a,b). In addition, Darhib talc deposits appeared with blue and red colors using MNF3, MNF1, MNF2, and MNF4, MNF1, MNF3 in RGB (Fig. 8c,d).

#### Band ratio (BR)

**Figure 9:**
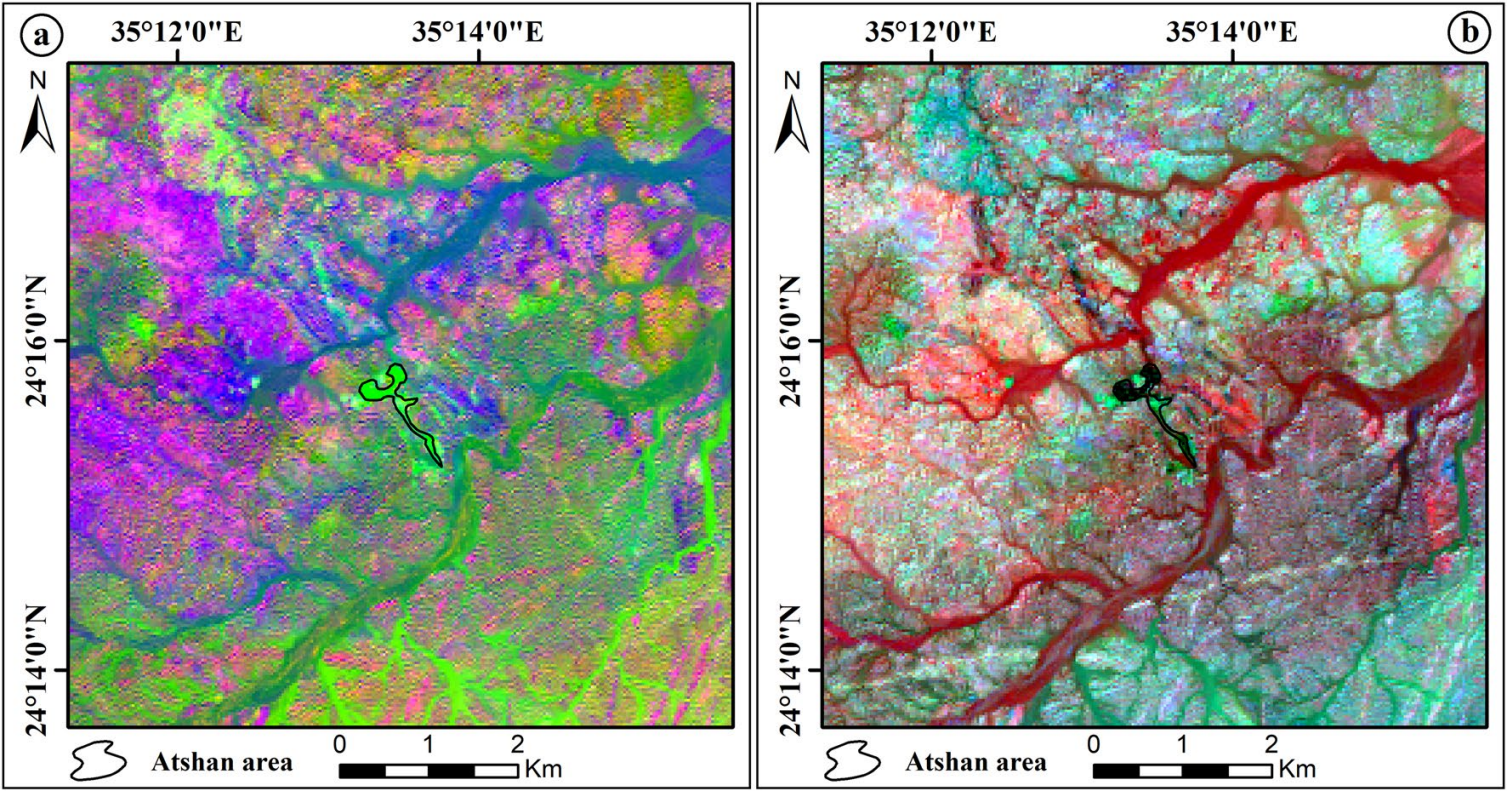
(**a**) OLI RGB color ratio image (6/7, 4/2 and 6/5) of Atshan area, and (**b**) OLI RGB color ratio image (5/4, 6/7 and 7/5) of Atshan area by using Arc GIS 10.4 and ENVI 5.3.

**Figure 10:**
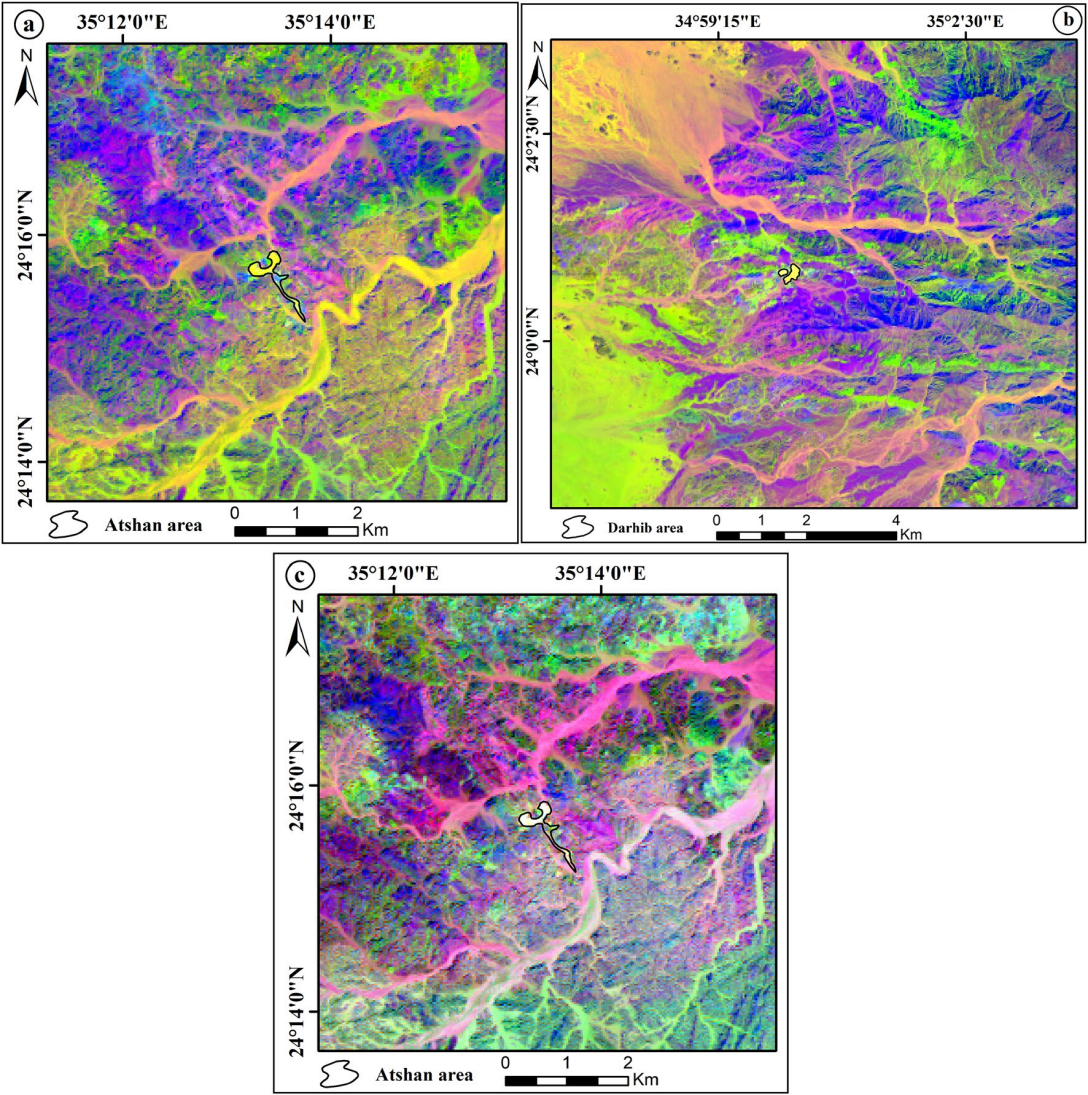
(**a**) OLI RGB color ratio image (2/4, 4/7 and 6/5) of Atshan area, (**b**) OLI RGB color ratio image (2/4, 4/7& 6/5) of Darhib area, and (**c**) OLI RGB color ratio image (4 + 3/5, 5/7, 2 + 1/3) of Atshan area by using Arc GIS 10.4 and ENVI 5.3.

Band-rationing is a common powerful image-processing technique in remote sensing, as it enhances the spectral differences between bands and highlights anomalies by dividing one spectral band by another^35^. Thus, this method was used in the current study to improve the ability to distinguish between rock units because ratioed images clearly show variations in the slopes of the spectral reflectance curves between the two bands involved, regardless of the absolute reflectance values observed in bands^36^. Several band ratios have been used in this study to discriminate talc deposits in the Atshan and Darhib areas.^37^ derived FCC image of three OLI-band ratios (6/7 in red (R), 4/2 in green (G), 6/5 in blue (B)) and the FCC (RGB- 5/4, 6/7, 7/5) that discriminate talc with light green and black colors, respectively of the Atshan area (Fig. 9a,b). Two new band ratios were proposed to differentiate talc deposits in the Atshan and Darhib areas, using the FCC ratios (2/4, 4/7, 6/5) and (4 + 3/5, 5/7, 2 + 1/3). The outcome of the first band ratio proposal illustrates the occurrence of talc deposits in the Atshan and Darhib areas, indicated by yellow pixels (Fig. 10a,b). The second band ratio focuses specifically on the talc resources in the Atshan region, represented by white pixels (Fig. 10c).

### Gravity data analysis

#### Regional and residual gravity data

**Figure 11:**
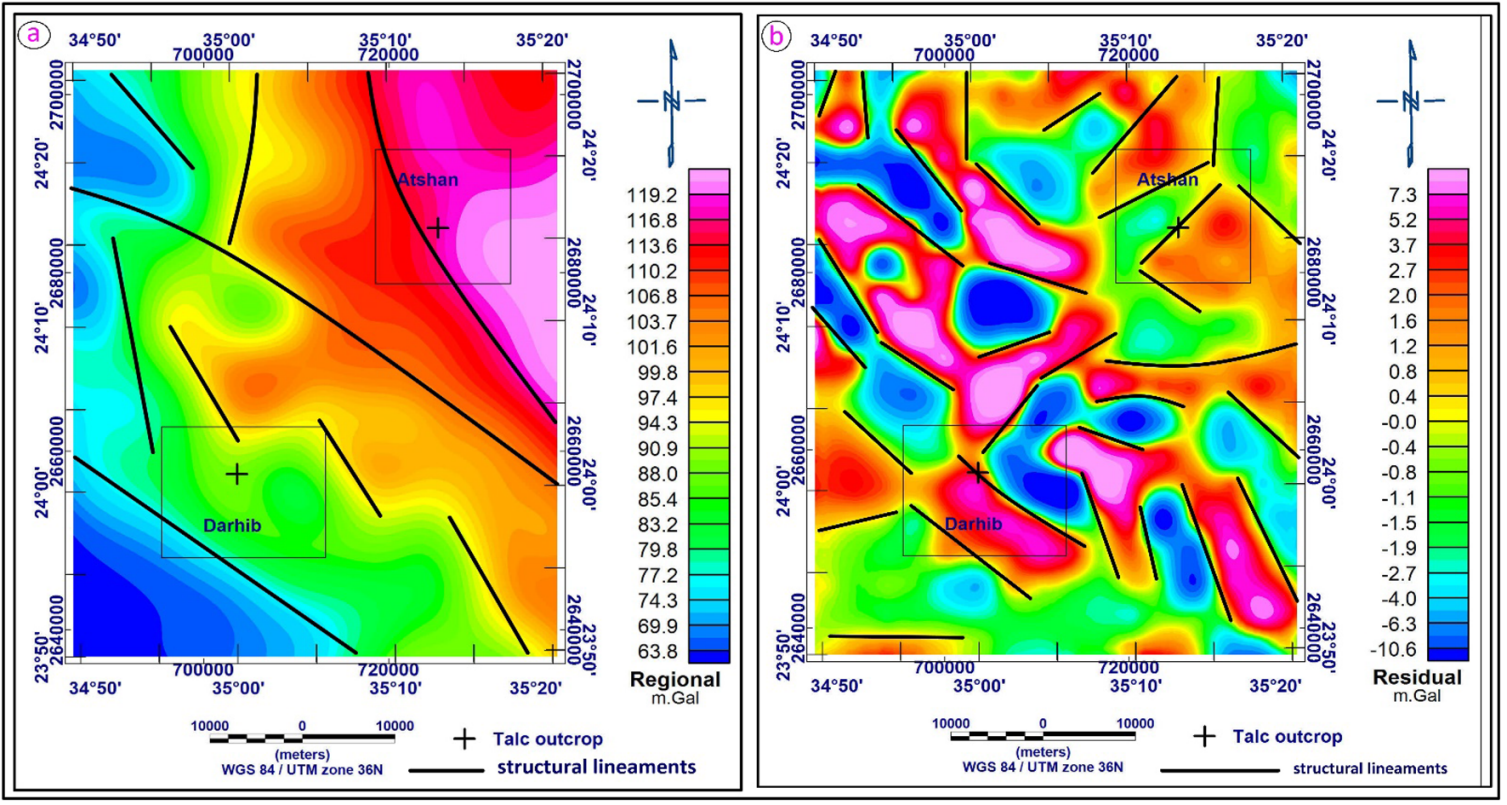
(**a**) Regional anomaly map of the study area displays regional structural lineaments; and (**b**) Residual anomaly map of the study area displays shallow structural lineaments (by using Oasis Montaj software version 8.3).

The gravity data is split into two components: the regional and the residual component maps. The regional component expressions are constituents of large-scale compositional variations within the buried crystalline basement complex (the intra-basement effect) and large-scale structural relief features (supra-basement effect). The deep (regional gravity) sources (Fig. 11a) are classified into two zones. The first zone is located in the northeastern part and is characterized by high gravity anomalies while the regional data value ranges between 99.8 and 119.2 m.Gal. Meanwhile, the second zone (low anomalies) covers the southwestern and western parts of the study area and is characterized by low gravity data ranging from 99.8 to 63.8 mGal. This means that the depth of basement rocks in the northeastern part is located near the earth's surface, while the southwestern and western parts are characterized by a thick sedimentary cover.

The residual map focuses attention on weaker features, which are obscured by strong regional effects on the original map^38^. The residual component comprises mainly the contributions of small-scale (detailed) structural features of the basement complex, in addition to the contributions of the potential intra-sedimentary sources (e.g., basaltic dykes and sheets). The most remarkable feature in the near-surface field (residual gravity) is the elongated anomaly in the various parts and trending NW–SE, NNW–SSE, NE–SW, and E–W bounding the large low regional zone. This may be due to a fault or contact structure in this area. Moreover, it is characterized by the presence of a system of narrow closures distributed all over the study area with local variations in both the amplitude and frequencies.

Structurally, the fault patterns at shallow and deep depths in the study area were interpreted from regional and residual maps. The NW–SE, NE–SW, and NNW–SSE trends are represented in the residual map (Fig. 11b), while the NW–SE is represented in the regional map (Fig. 11a). It means that the NW–SE trend is the deepest one in the prospective area of study. Meanwhile, the other trends related to near-surface structures in the prospect area of study.

#### Analytic signal technique

**Figure 12:**
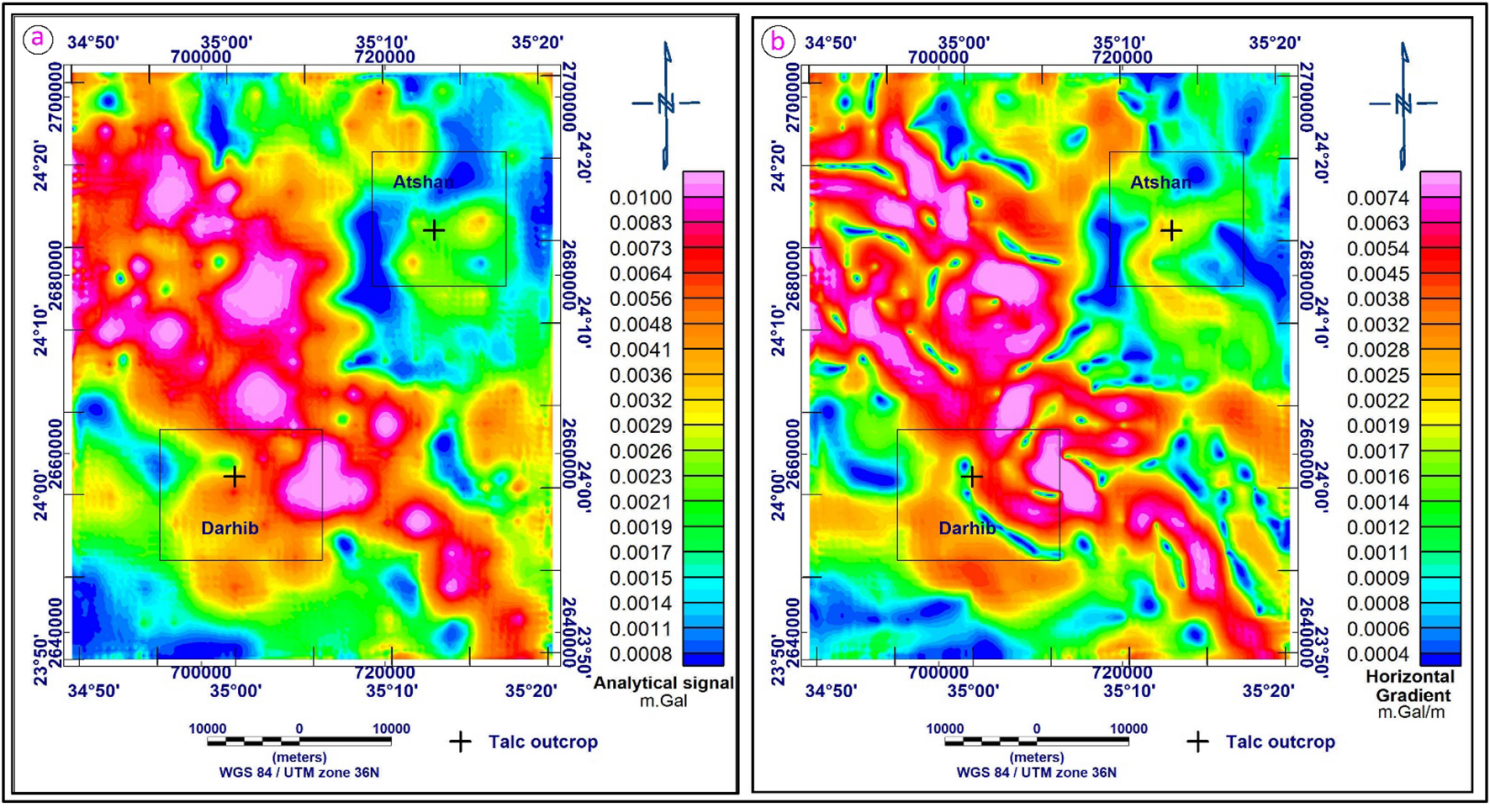
(**a**) Analytical signal anomaly map of the study area; (**b**) Horizontal gradient anomaly map of the study areas (by using Oasis Montaj software version 8.3).

The analytical signal (AS) technique represents the envelope of both horizontal and vertical derivatives over all possible directions of the earth's gravitational field. It’s independent of the strike and dip of perturbing gravity anomalies as well as the direction of interest. By transforming the analytical signal (AS), anomalies are placed right above their appropriate causal bodies. This is achieved by differentiating the total regional field gradient in three perpendicular directions at each measurement point. The mathematical basis of this transformation technique is detailed in this manuscript^39,40^. The analytical signal of the Bouguer anomaly map (Fig. 12a) defines many high features, especially in the northwest and extending to the southeast corner of the map, which is associated with variations in stratigraphic thickness as well as the lithological changes within the basement itself. The anomaly patterns on the analytical signal map reveal that the area is dominated by the NW–SE and N–S anomalies zones, which represent a particular gravity signal of the Gulf of Suez-Red Sea Clysmic System. It is also distinguished by a strong gradient in the NW direction associated with changes in the basement rocks. Locations and, perhaps, trends of maximum density contrast are frequently linked with petrophysical changes within the crystalline basement complex. The Atshan area is characterized by intermediate to low analytical signal values ranging between 0.0032 and 0.0008 mGal/m, while the talc occurrence in the Atshan area is characterized by an intermediate value. On the other hand, the Darhib area is located in the southern part of the map. This region is characterized by AS values ranging from low to high (0.0008–0.0100 mGal/m). The intermediate to low values are probably anomaly signatures from sedimentary infills. The talc appearance in this area is characterized by intermediate AS values.

#### Horizontal gradient (HG) technique

The horizontal gradient approach has been extensively utilized to determine density boundaries using gravity data. If the boundaries of a tabular body are vertical and widely spaced from each other, the horizontal gradient of the gravity anomaly induced by the body tends to overlie the edges^41^. In comparison to the vertical gradient approach, which is primarily beneficial for detecting shallower structures, the method is also more robust in defining both shallow and deep sources. The horizontal gradient transformation tends to have extreme values on the potential field signal's steepest slope and enhances higher frequencies.

The horizontal gradient is helpful in edge identification because of these characteristics. The frequency domain calculation of the amplitude of the horizontal gradient for the research area's gravity data (Fig. 12b). Generally, this map utilizes a horizontal gradient technique to identify the edges and boundaries of the body, with high anomalies (red color) indicating those locations. The HDR map of the study area shows values ranging from 0.0004 to 0.0074 mGal/m. The high values of HDR are mainly located in the northwestern and extend to the southeastern part of the study area. The locations where talc appears in the Atshan region on the Horizontal Gradient map are characterized by intermediate to high values, and the major trend of anomaly is NE–SW. This means that this area was affected by structure in the direction of northeast-southwest. The talc in the Darhib area is characterized by high HG values and the general direction of the anomaly is NW–SE. The maxima in HG also represent the geological contacts; the interpreted contacts from HG match known geological contacts.

#### Source parameter imaging (SPI) technique

The Source Parameter Imaging (SPI) approach is a mechanism for automatically calculating the source depths from gridded gravity data^42^. The formula below applies to calculate the source parameter imaging depth.

1 $${\text{k}} = \frac{{\frac{{\partial^{2} {\text{M}}}}{{\partial {\text{x}}\partial {\text{z}}}}\frac{{\partial {\text{M}}}}{{\partial {\text{x}}}} - \frac{{\partial^{2} {\text{M}}}}{{\partial {\text{x}}^{2} }}\frac{{\partial {\text{M}}}}{{\partial {\text{z}}}}}}{{\left( {\frac{{\partial {\text{M}}}}{{\partial {\text{x}}}}} \right)^{2} + \left( {\frac{{\partial {\text{M}}}}{{\partial {\text{z}}}}} \right)^{2} }}$$

2 $$\mathrm{Depth}=\frac{1}{{\mathrm{K}}_{\mathrm{max}}}$$

where K_max_ is the maximum value of the local number K that is measured across the step source and M is the total gravitational field.

**Figure 13:**
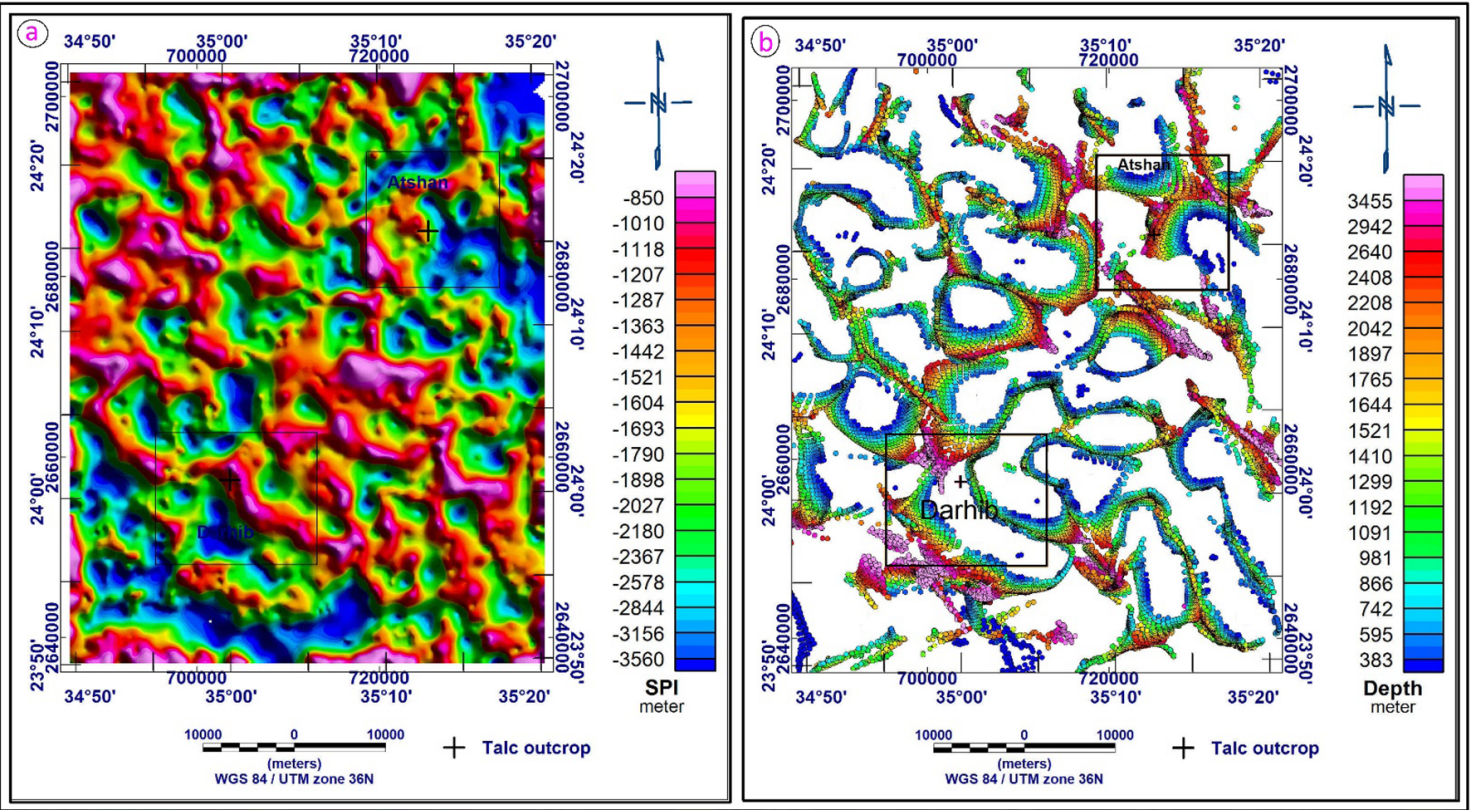
(**a**) Source parameter imaging map of the study area, (**b**) 3D Euler deconvolution of the study area for structural index (by using Oasis Montaj software version 8.3).

The source parameter Imaging map of the research region (Fig. 13a) depicts a large basement structure swell marked by red color. This swell extends from the southwest corner to the northeast corner of the study area. The average depth of the swell is 850 m. This swell is bounded by a blue-colored deep basement structure (trough). The average depth of the trough reaches 3560 m, respectively. The southeastern and northwestern parts of the Atshan have deep basement depths, whereas the central part has shallow basement depths that extend from the northeastern to the southwestern corner. Meanwhile, the depth of the basement in the Darhib area increases directly from the southwestern and northwestern parts to the central part of the Darhib area.

#### Euler deconvolution (ED) technique

Euler deconvolution has been well established and widely used as an interpretation method to estimate source location and depth from potential fields (gravity and magnetic) for various geological sources such as dikes, faults, contacts, and extrusive^43^. In recent years, the Euler method has gained considerable popularity in environmental applications^21,44^. Based on the previous analysis, Euler deconvolution has been employed to determine structural index and depth simultaneously for simple sources. The horizontal location is determined using the conventional Euler method by applying approximated structural index value.

The results of applying the Euler deconvolution technique (structural index = 0) to the gravity anomaly map are represented in a three-dimensional perspective view of the structure of the basement rocks map for the study area (Fig. 13b). This map displayed useful information about the structural framework of the study areas and provided a clear picture of basement features. Generally, the structural pattern (faults and similar structural features) acts as a controlling feature on the ore deposition and has a bearing on the depths of the ore deposits. Most of the shallow and deep contacts extend in the NW–SE and NE–SW directions. Therefore, most of these lineaments coincide with the surface lineament distribution traced from the geological map of the area. The obtained depth values by using a structural index equal to zero range from less than 383 m to more than 3455 m.

## Discussion

New geochemistry has been done for both talc deposits collected from Atshan and Darhib areas to manifest their chemical composition (major and trace elements) and deduce their origin. In addition, we used remote sensing data and gravity data techniques to detect their extension and depth, respectively. This reflects a large reserve of talc deposits in the examined areas that is widely used in several industrial applications.

### Remote sensing data analysis

Several remote sensing techniques have been used for lithological mapping of the research area using Landsat-8 data. Based on the OIF ranking of Landsat-8 image, the best FCC for talc deposit discrimination is (RGB-7, 5, 3). As well as the MNF3, MNF1, MNF2, and MNF4, MNF1, MNF3 gave the best representation of talc deposits in the Atshan and Darhib areas. Moreover, the best PCA (PC1, PC2, PC3, and PC4, PC2, PC1) in RGB showed good discriminates of talc deposits in the Atshan area. In addition to Darhib talc deposits the color composite images (PC3, PC2, PC1, and RGB- PC4, PC3, PC1) RGB have been produced to separate talc deposits. Moreover, two new FCC band ratios (2/4, 4/7, 6/5) and (4 + 3/5, 5/7, 2 + 1/3) have been derived to distinguish the talc deposits in two case studies, Atshan and Darhib areas.

### Gravity data analysis

Gravity anomalies can be caused by local rock types that vary enough in density to be detectable. Examples include sedimentary rocks and their weathered derivatives that fill basins, which are typically characterized by gravity lows on anomaly maps due to the prevalence of quartzo-feldspathic minerals. High gravity is commonly correlated with mafic and basic rocks because of the abundance of Fe–Mg minerals inside them. These variations are useful for inferring structures like basins, arches, and buried intrusions, mapping huge areas where rocks are inaccessible or obscured, and searching for faults responsible for juxtaposing rocks of different densities.

To provide a qualitative and quantitative interpretation of the gravity data, several techniques such as regional residual horizontal gradient (HG), analytic signal (AS), source parameter imaging (SPI), and Euler deconvolution (ED) were used. According to the analyses, the predominant directions of the structural lineaments are northwest to southeast, northeast to southwest, and north-northwest to south-southeast. The depth result from the SPI varies from 850 m for shallow sources to 3560 m for sedimentary thickness, while the depth estimate from the 3D Euler deconvolution indicated that the depths to the sources ranged from 383 to 3453 m.

### Origin of talc deposits

The geochemical characteristics of the investigated talc deposits suggest mantle ultramafic protolith. This can be inferred from their high MgO (av. 27.89 wt.%), Cr ( av. 675 ppm), Co ( av. 50 ppm), and Ni (av. 1252 ppm) contents and CaO depletion^8,10,26,45^. Some major oxides, such as SiO_2_ and Al_2_O_3_ are relatively immobile during variable alterations^46^. In addition, the Al_2_O_3_/SiO_2_ ratio can be used to separate variable tectonic regime^47^. The examined talc protolith is mainly ultramafic rocks with MgO (av. 27.89 wt.%) and Fe_2_O_3_ (av. 5.33 wt.%), suggesting the nature of depleted mantle^48^, which is emplaced in a forearc setting (Fig. 5d). The examined samples have low Al_2_O_3_ (av. 0.72) and SiO_2_/MgO (av. 2.15) contents, which is comparable with ophiolitic peridotite (Fig. 5e). This is indicated by the low contents of CaO (av. 0.32 wt.%), TiO_2_ (av. 0.04 wt.%), and Al_2_O_3_ (av. 0.72 wt.%). In addition, they have low contents of Al_2_O_3_/SiO_2_ and MgO/SiO_2_, which are lower than those of the primitive mantle (~ 0.1 and ~ 0.85, respectively)^29,49^.

### Source of hydrothermal fluids

**Figure 14:**
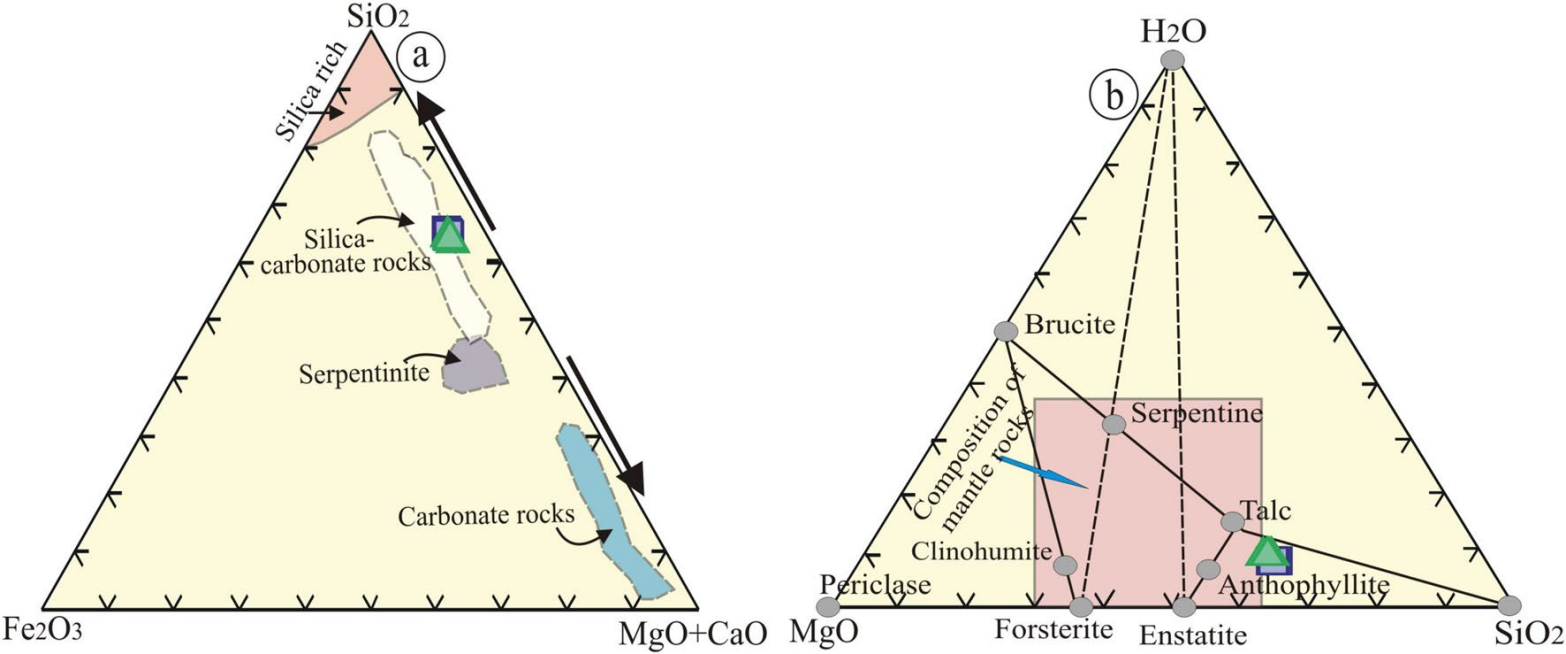
(**a**) SiO_2_–Fe_2_O_3_–(MgO + CaO) ternary diagram. Fields of serpentinites, silica- carbonate, carbonate-rich, and silica-rich rocks^7,50,51,60^; and (**b**) H_2_O-SiO_2_-MgO ternary diagram^7^ (by using Coreldrow program version 2012).

Talc, carbonates, chlorite, and serpentine are the main metasomatic mineral phases, which reflect the metasomatic processes of mantle rocks^6,7,50,51^. These processes are responsible for MgO, Ni, Cr, and Co enrichment in comparison to PM^49^. The examined samples plot into silica carbonate compositional fields (SiO_2_–Fe_2_O_3_–(CaO + MgO) diagram), which are identified by mineralogical compositions. This field represents a transition zone between silica-rich and serpentinite fields (Fig. 14a). By using the ternary diagram of H_2_O–SiO_2_–MgO ^7^, the examined samples plot between the anthophyllite and talc lines, reflecting their typical mineralogical constituents (Fig. 14b).

The dominant mantle rocks are enriched with ferromagnesian minerals that can be replaced by metasomatic minerals due to the infiltration of CO_2_/H_2_O fluids, reflecting the overprint of greenschist facies metamorphism^49,52^. These metasomatic minerals are widely abundant along shear zones and fault planes (certainly with ophiolitic rocks due to high deformation processes), suggesting the role of hydrothermal solutions. These shear zones acted as channels for hydrothermal mineralizing fluids that are responsible for talc deposits. Furthermore, these zones may have been formed as a result of tectonic faulting, which led to ultramafic and metavolcanic combinations.

These fluids probably resulted from dehydration during metamorphism (early stage) or magmatic fluids from nearby granitic intrusions (late stage) certainly along from W. Reiidi (Atshan) and El Kharit (Darhib) granitic rocks that are responsible for silica additions^4,53^.

In addition, hydration of anhydrous mantle minerals can produce talc mineral^54^;

$${\text{2Mg}}_{{2}} {\text{SiO}}_{{4}} + {\text{ 3H}}_{{2}} {\text{O }} \to {\text{ Mg}}_{{3}} {\text{Si}}_{{2}} {\text{O}}_{{5}} \left( {{\text{OH}}} \right)_{{4}} + {\text{ Mg}}\left( {{\text{OH}}} \right)_{{2}}$$

During Si-metasomatism of hydrous minerals, talc can be formed at greenschist facies to lower amphibolite facies (> 300–400 °C), which led to a decrease of MgO/SiO_2_ ratios^55^.

$${\text{Serpentine }} + {\text{ 2SiO}}_{{2}} = {\text{ talc }} + {\text{ H}}_{{2}} {\text{O}}$$

On the other hand, talc can be formed by serpentinization of mafic (basaltic) rocks by metamorphic processes (hydrothermal solutions rich in silica and magnesium) forming metasomatic minerals^56^.

$$\begin{aligned} & {\text{2CaMgSi}}_{{2}} {\text{O}}_{{6}} + {\text{ 3 Mg}}_{{2}} {\text{Si}}_{{2}} {\text{O}}_{{6}} + {\text{ 3H}}_{{2}} {\text{O }} \to {\text{ Ca}}_{{2}} {\text{Mg}}_{{5}} {\text{Si}}_{{8}} {\text{O}}_{{{22}}} \left( {{\text{OH}}} \right)_{{2}} + {\text{Mg}}_{{3}} {\text{Si}}_{{2}} {\text{O}}_{{5}} \left( {{\text{OH}}} \right)_{{4}} \\ & {\text{Diopside}}\,\,\,\,\,\,\,\,\,\,\,\,\,\,\,\,\,\,\,\,\,\,\,\,\,\,\,\,{\text{Enstatite}} \;\;\;\;\;\;\;\;\;\;\;\;\;\;\;\;\;\;\;\;\;\;\;\;{\text{Tremolite}}\;\;\;\;\;\;\;\;\;\;\;\;\;\;\;\;\;\;\;\;\;\;\;\;{\text{Serpentine}} \\ \end{aligned}$$

Furthermore, late-stage alteration of mafic rocks causes alterations represented by silicification, chloritization, and epidotization^4^. This stage is mainly responsible for sulphide-bearing talc-rich rocks along shear zones^4^. According to Schandl et al.^13^, there are series of metamorphic assemblages ranging from contact metamorphism (between magmatic intrusion and volcanic rocks) to greenschist facies.

From the previous, we can conclude that the talc deposition may be related to greenschist facies metamorphism or to a magmatic solution that is (associated with granitic intrusions) interacting with the surrounding volcanic rocks and forming metasomatic minerals.

## Conclusion

The combined mineralogical and geochemical detailed studies of talc deposits collected from Atshan and Darhib, south Eastern Desert, Egypt, reveal that they are comparable with the ultramafic ophiolitic peridotite. They are structurally controlled, occurring as lenses or pocket bodies following NNW–SSE and E–W directions in Darhib and E–W in the Atshan area. They are enriched in incompatible elements and possess positive anomalies of semi-volatile elements such as Pb and strong negative anomalies of LFSEs such as Sr. In addition, clear pronounced positive As, Sn, and Cd anomalies, which is attributed to the abundance of sulfide minerals. Detecting talc deposits in the Darhib and Atshan areas was successfully accomplished using several remote sensing techniques, i.e., false color composite (FCC), principal component analysis (PCA), minimum noise fraction (MNF), and band ratio (BR), which were applied to Landsat-8 data. A new two FCC band ratios were created to distinguish talc deposits of Atshan and Darhib areas. The gravity data were qualitatively interpreted using regional, residual horizontal gradient (HG) and analytical signal (AS) techniques. The quantitative interpretation was done using source parameter imaging and Euler deconvolution methods. The results showed that the trends of the structural lineaments dominate in the NW–SE, NE–SW, NNW–SSE, and E–W directions. The SPI depth result ranges from 850 m for shallow sources to 3560 m for sedimentary thickness, and the 3D Euler deconvolution depth estimate revealed depths to sources ranging from 383 to 3453 m. The results of these techniques are very close to each other. We suggest that the talc deposits may be related to regional metamorphism or to magmatic fluids that are (associated with granitic intrusions) interacting with the surrounding volcanic rocks and forming metasomatic minerals.

## Data Availability

All data generated or analyzed during this study are included in this manuscript.
